# Assessing volume status in heart failure: The role of renal duplex ultrasound in evaluating cardiorenal morbidity and heart failure mortality

**DOI:** 10.2478/jccm-2025-0029

**Published:** 2025-07-31

**Authors:** Mohamed Elsayed Elrokh, Waleed Shehata Hassan, Ramadan Ahmed Khalil, Ayman Nehad Moharam, Emad Eldin Omar Abdelaziz

**Affiliations:** Department of Critical Care Medicine, Faculty of Medicine, Cairo University, Cairo, Egypt

**Keywords:** heart, failure, cardiorenal, renal, duplex, and venous flow pattern

## Abstract

**Background:**

Critical care physicians face challenges managing decompensated heart failure. This study aims to examine the volume status of patients with decompensated heart failure and evaluate the effectiveness of the renal resistive index (RRI) and renal venous flow pattern (VFP) in assessing volume status and predicting outcomes related to cardiorenal syndrome and mortality.

**Patients and methods:**

This prospective study was conducted in the intensive care unit of Kasr Elainy Hospital at Cairo University with patients admitted for acute decompensated heart failure (ADHF). Patients were subjected to clinical screening, laboratory measurements, and echocardiographic examination, including cardiac index renal duplex.

**Results:**

This study included 61 patients with a mean age of 64.8±9.1 years. Renal duplex parameters were 0.692±0.087 for the mean RRI, and the percentages of VFP were as follows: continuous 49.2%, biphasic 27.9%, and monophasic 23%. Elevated proBNP levels and IVC collapsibility index were significantly associated with RRI ≥0.75 and abnormal VFP patterns in assessing volume status. The ROC curve of the RRI, VFP, proBNP, SOFA score, ADHERE risk score, and GWTG-HF score for AKI occurrence showed that RRI has 68% sensitivity to detect AKI, but VFP has better results with 86.4% sensitivity. RRI has a prognostic role in predicting in-hospital mortality in acute heart failure, as RRI has 83.3% sensitivity, and VFP showed better results with 83.3% sensitivity. Also, VFP had a better predictive value for the incidence of 3 months mortality with 90.9% sensitivity, while RRI has 63.4% sensitivity.

**Conclusion:**

Renal duplex measures, such as VFP and RRI, are highly effective prognostic tools for identifying worsening renal function. Beyond renal outcomes, these measures also serve as reliable predictors of mortality and survival in patients with acute decompensated heart failure, offering clinicians the opportunity to tailor therapeutic approaches early during treatment.

## Background

Acute decompensated heart failure (ADHF) is characterized by a sudden exacerbation of heart failure symptoms, typically resulting from cardiogenic pulmonary edema due to fast fluid accumulation in the lungs [1]. The predominant symptoms and indicators of ADHF are directly associated with intravascular congestion, which may arise from the gradual accumulation of fluid by interdependent pathways [2]. This encompasses sodium retention attributable to renal impairment or noncompliance with medication regimens, elevated left ventricular filling pressures leading to heightened pulmonary and central venous congestion, and fast central redistribution of intravascular volume from peripheral or splanchnic venous circulation [3]. The supplementary preload raises end-diastolic pressures, amplifying ventricular wall stress and myocardial oxygen demand, hence exacerbating diastolic function [4]. Expanded ventricular volumes can produce or exacerbate functional tricuspid or mitral regurgitation, further increasing venous pressures, which can worsen renal function.

Precise fluid status assessment in ADHF is essential, as persistent congestion directly worsens outcomes [5]. This requires integrating clinical evaluation (edema), biomarkers (BNP/NT-proBNP, hemoconcentration), and imaging (lung ultrasound B-lines, echocardiographic IVC dynamics) [5]. However, mechanical ventilation (MV), particularly positive end-expiratory pressure (PEEP), significantly alters this assessment and underlying hemodynamics [6]. PEEP minimizes systemic venous return, significantly decreasing preload to the preload-dependent right ventricle (RV). Simultaneously, higher intrathoracic pressure increases RV afterload by compressing the pulmonary vasculature and raising pulmonary vascular resistance [7,8]. While LV afterload reduction may offer a transient advantage, this dual effect on the RV—preload reduction and afterload elevation—risks acute RV failure, especially with pulmonary hypertension [9]. Consequently, MV obscures fluid status interpretation, demanding intensified multimodal monitoring.

Cardiorenal syndrome (CRS) is a recognized and complex clinical disease that illustrates the interplay between the heart and the kidneys [10]. CRS Type 1 (CRS1) is characterized by the decline of cardiac function, such as sudden heart failure, resulting in acute kidney damage (AKI) [11]. Approximately 25–44% of patients with acute heart failure will get CRS1 during hospitalization, which is closely associated with extended hospital stays. The negative clinical results underscore the necessity of identifying patients at high risk for acquiring CRS1[12]. Diagnosing CRS1 predominantly relies on blood creatinine and urine output alterations, which have demonstrated insufficient sensitivity, leading to suboptimal clinical results [13].

The Doppler-derived renal resistive index has been utilized for years in multiple clinical contexts, including the evaluation of chronic renal allograft rejection, the detection and management of renal artery stenosis, differential diagnosis of acute and chronic obstructive renal disease, and the prediction of renal and global outcomes in critically ill patients [14,15]. Evidence indicates that an elevated renal resistive index signifies alterations in intrarenal perfusion and is also associated with systemic hemodynamics and subclinical atherosclerosis [15,16].

This study seeks to examine the pathophysiological relationship between renal microcirculation and the cardiovascular system. It highlights the patient’s overall condition prior to analyzing the renal duplex parameters and evaluates the effectiveness of renal duplex parameters in monitoring decongestive strategies during the management of ADHF. The objective is to categorize patients based on their RRI and renal venous flow pattern (VFP) and ascertain the mortality and morbidity associated with those who develop cardiorenal syndrome.

## Methods

### Patients

This prospective clinical trial was performed on sixty-one patients admitted to the Intensive Care Unit (ICU) of the Critical Care Department at Kasr-Alainy Hospitals, Cairo University, from October 2022 to January 2024. The local Ethics Committee accepted the study protocol on September 23, 2022, with approval number MD_284_2022.

**Inclusion criteria:** Cases admitted to the ICU diagnosed with ADHF as defined according to the 2021 ESC guidelines [17] and confirmed consent from every patient aged 18 years or older.

**Exclusion criteria**: Individuals under 18 years, patients diagnosed with cardiogenic shock already on an intra-aortic balloon pump (IABP) or extracorporeal membranous oxygenation (ECMO), those with terminal advanced diseases, patients undergoing hemodialysis, pregnant individuals, patients with severe stenotic valvular heart disease, patients with renal conditions that could influence renal blood flow measurements, and patients having intraperitoneal pressure exceeding 12 mm Hg.

The sample size of 61 was calculated using PASS software (PASS 11). NCSS, LLC. Kaysville, Utah, USA) [18]. It is rationalized by the study’s objective to detect a significant difference in proportions from a null hypothesis of 0.5 to an alternative hypothesis of 0.9, considering a prevalence of 20%. This calculation aims for a power of 90%, which is crucial for minimizing Type II errors, thereby ensuring that meaningful effects are not overlooked [19]. The significance level of 0.05 is standard in the field, reinforcing the validity of our findings. While the sample size may seem modest, it reflects practical constraints such as resource availability and participant recruitment challenges. Furthermore, the choice of N1 as 12 can be seen as an initial exploratory phase, allowing for adjustments based on preliminary results [20,21]. This approach aligns with similar studies in the literature [22,23,24,25,26], making it a reasonable and feasible strategy to address the research question while acknowledging the limitations inherent in studying low-prevalence outcomes [27].

### Clinical and laboratory assessments

Baseline demographic and clinical data, including medical history, were recorded. Blood samples were collected at admission to analyze renal function, including serum creatinine, blood urea nitrogen, and glomerular filtration rate (GFR). GFR was calculated GFR was calculated using the MDRD formula: GFR (ml/min/1.73m^2^)=186×(serum creatinine)−0.154×(age)−0.203×(0.742 if female)×(1.21 if black);. Volume status markers such as proBNP were also measured (Rule-in values for the diagnosis of acute HF: >450 pg/mL if aged <55 years, >900 pg/mL if aged between 55 and 75 years, and >1800 pg/mL if aged >75 years). Additional laboratory assessments included electrolytes, hemoglobin and Liver function test.

### Echocardiography, Doppler ultrasonography, and renal duplex assessment

Transthoracic echocardiography was performed using a GE Vivid E9 system with a sector transducer (2.5–5 MHz). Key assessments included left ventricular ejection fraction, Left ventricular end-diastolic diameter, Left ventricular end-systolic diameter, Cardiac index, Pericardial effusion (presence and severity), Mitral regurgitation (presence and severity), Tricuspid regurgitation (presence and severity).

Renal Doppler imaging was performed with patients in the supine or left lateral decubitus position, and typically, only the right kidney was assessed. The assessments were conducted using a Philips EPIQ 7 ultrasound system equipped with a 2–5 MHz curvilinear transducer. Interlobar renal vessels were identified using color Doppler with aliasing velocity set to 15 cm/s. Blood flow was interrogated using pulsed-wave Doppler during held respiration, with care to ensure parallel alignment between the direction of interlobar vessel flow and the ultrasound beam sample volume. The pulsed-wave Doppler velocity scale was set to 15–30 cm/s and the wall filter to a minimum. Arterial and venous flow signals were recorded simultaneously. For patients with irregular cardiac rhythm, measurements were performed using an index cardiac cycle, the cardiac cycle following a preceding and pre-preceding R-R interval of similar duration. The renal duplex assessment included measuring peak systolic velocity and end-diastolic velocity and calculating the renal resistive index (RRI) using the formula: [(peak systolic velocity – end-diastolic velocity)/peak systolic velocity]. Venous flow patterns (VFP) were evaluated and categorized into continuous, biphasic, or monophasic patterns. In addition, renal interlobar venous impedance indices were assessed to evaluate venous congestion.

The inferior vena cava (IVC) collapsibility index was also assessed using a subxiphoid approach. Measurements of IVC diameter during inspiration and expiration were taken, and the IVC collapsibility index was calculated as [(IVC diameter on expiration - IVC diameter on inspiration) / IVC diameter on expiration] × 100. An index >50% indicated significant collapsibility, reflecting intravascular volume depletion. Each parameter was averaged over three cardiac cycles to ensure accuracy.

### Data Collection and Grouping

Patients were stratified based on RRI values (≥0.75 or <0.75) and VFP patterns (continuous, biphasic, monophasic). Associations between these parameters and clinical outcomes, including AKI, in-hospital mortality, and 3-month readmission or mortality, were evaluated.

### Clinical Outcome

The primary clinical outcomes evaluated in this study included acute kidney injury (AKI), in-hospital mortality, and three-month all-cause mortality or rehospitalization. AKI was defined using the KDIGO (kidney disease: Improving Global Outcomes) criteria as an increase in serum creatinine by ≥0.3 mg/dL within 48 hours or a 1.5-fold increase from baseline within seven days. Mortality and readmission events were recorded and verified through hospital records and follow-up interviews. Secondary outcomes included the duration of hospital stay, changes in renal function, and the need for renal replacement therapy during hospitalization. The association of these outcomes with renal resistive index (RRI) and venous flow patterns (VFP) was analyzed to determine their prognostic significance in patients with ADHF.

Prognostic scores were calculated for the enrolled patients, including SOFA, the Acute Decompensated Heart Failure National Registry (ADHERE), and GWTG-Heart Failure Risk. Additionally, all patients adhered to a diuretic protocol and were managed according to the guidelines for the management of ADHF (Guidelines for the Diagnosis and Management of Heart Failure in Adults) [28]. Treatment objectives focused on preventing organ dysfunction by improving symptoms, maintaining SBP >90 mmHg and peripheral perfusion, and sustaining SpO_2_ >90%.

### Statistical Analysis

Data were encoded and input utilizing SPSS version 28 (IBM Corp., Armonk, NY, USA). Data were summarized utilizing means, standard deviations, medians, minimums, and maximums for quantitative variables, alongside frequencies (case counts) and relative frequencies (percentages) for categorical variables. Group comparisons were conducted using unpaired t-tests or analysis of variance (ANOVA) with post hoc multiple comparisons for normally distributed quantitative variables, whereas the non-parametric Kruskal-Wallis test and Mann-Whitney test were employed for non-normally distributed quantitative variables [29]. The Chi-square (χ^2^) test was carried out to compare categorical data [30]. The exact test was utilized when the expected frequency was below 5. Correlations among quantitative variables were evaluated using the Spearman correlation coefficient [31]. The ROC curve was constructed, and a study of the area under the curve was conducted to determine the appropriate cutoff value of significant parameters for detecting different outcomes. Survival curves were generated utilizing the Kaplan-Meier method and evaluated with the log-rank test. P-values were deemed significant if < 0.05.

## Results

This study included 61 patients; 55.7% were males, and 44.3% were females, with a mean age of 64.8±9.1 years and an average BMI of 24 ± 3.2 kg/m^2^. The most prevalent comorbidities among the study population were hypertension (72.1%), ischemic heart disease (60.7%), diabetes mellitus (50.8%), atrial fibrillation (AF) (32.8%), and COPD (36.1%). Five patients had a history of implantation cardiac device divided a follow (1 with VVI, 3 with DDD, and 1 with CRT.P). 55.7% of the patients had ACEI drugs on their own medications list, while 57.4% of them had beta-blockers and 60.7 % had diuretics.

The clinical examination revealed that 54.1% of patients were in heart failure New York Heart Association (NYHA) class IV upon admission, while 14.8% were in cardiogenic shock. The mean systolic blood pressure was 125.1±26.7 mmHg, and the diastolic blood pressure was 71.6±23.25 mmHg, with a mean central venous pressure of 15.3±4.6 cm.H_2_O and mean arterial blood pressure of 89.6±22.7 mmHg. Upon examination, 63.9% of patients exhibited lower limb edema and 62.3% presented with pulmonary rales on auscultation.

Renal duplex parameters were 0.692±0.087 for the mean renal resistive index, and the percentages of venous flow patterns were as follows: continuous 49.2%, biphasic 27.9%, and monophasic 23%. The mean EF was 43.5±9.8, the mean CI was 2.8±0.54, and the mean percentage of the inferior vena cava collapsibility index was 23.08±6.08%. During the follow-up, 36.1% of patients developed AKI while in the hospital. The mean length of hospital stay was 6.5±2.5 days. During the hospital stay, 9.8% of patients died. In the 3-month follow-up, 32.8% of patients were readmitted to the hospital due to cardiovascular decompensation, and 18% of them died during this follow-up period.

### Correlation between RRI, VFP, parameters of volume status of patients

**Fig. 1.**
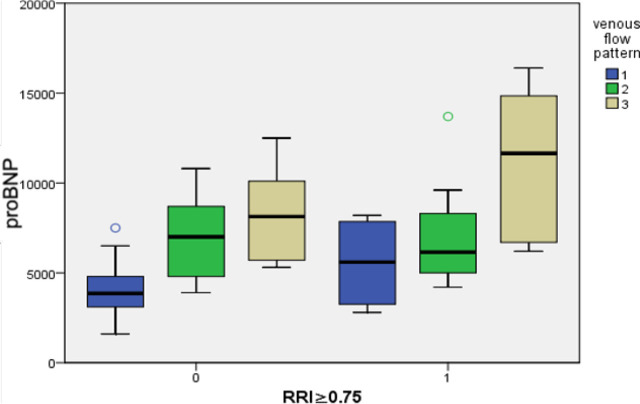
Correlation between RRI, venous flow pattern, and proBNP RRI≥0.75 (0=no, 1=yes) VFP (1=Continuous, 2= biphasic, 3=monophasic)

Table 1 represents the correlation between RRI, VFP, and parameters of the volume status of patients. By clinical examination, RRI correlates only with central venous pressure (P-value of 0.045), indicating a clinical sign of volume overload. In contrast, the VFP correlates with all studied parameters, including NYHA class (P-value of < 0.001), central venous pressure (p-value of < 0.001), lower limb edema (P-value of 0.005), presence of the third heart sound (P-value of 0.002), and presence of pulmonary rales (P-value of 0.032). Through radiological examination, RRI correlates with chest X-ray findings (P-value of 0.004) and the IVC collapsibility index (P-value of < 0.001). Additionally, the VFP correlates with chest X-ray findings (P-value of < 0.001) and the IVC collapsibility index (P-value of 0.019). Laboratory investigations reveal that RRI correlates with proBNP (P-value of 0.002) and the PaO_2_/FiO_2_ ratio (P-value of 0.043). Furthermore, the VFP correlates with proBNP (P-value of < 0.001) and the PaO_2_/FiO_2_ ratio (P-value of < 0.001). As shown in Figure 1, we assessed the complex relationship between RRI, venous flow pattern, and proBNP, observing that at higher RRI values above 0.75 and greater grades of venous congestion (grade 3 monophasic), proBNP shows the highest values, indicating increased volume overload. However, when RRI values exceed 0.75, but the grades of venous congestion are lower, proBNP exhibits lower values, suggesting decreased venous congestion.

**Table 1.** Correlation between RRI, venous flow pattern, and parameters of volume status of patients

**Parameters**			**RRI≥0.75**			**Venous flow patter**			

			**Yes (n=20)**	**No (n=41)**	**P-value**	**Continuous (n=30)**	**Biphasic (n=17)**	**Monophasic (n=14)**	**P-value**
New York heart association class		2	n=1 (5%)	n=3 (7.3%)		n=4 (13.3%)	n=0 (0.0%)	n=0 (0.0%)	
		3	n=6 (30%)	n=18 (43.9%)	0.622	n=17 (56.7%)	n=7 (41.2%)	n=0 (0.0%)	< 0.001
		4	n=13 (65%)	n=20 (48.8%)		n=9 (30%)	n=10 (58.8%)	n=14 (100%)	

Central venous pressure (cmH_2_o)			17.00±4.08	14.49±4.6	0.045	12.20±3.32	17.41±4.00	19.43±2.79	< 0.001
Lower limb edema			n=12 (60%)	n=27 (65.9%)	0.655	n=17 (56.7%)	n=8 (47.1%)	n=14 (100%)	0.005
3rd heart sound			n=5 (25%)	n=3 (7.3%)	0.100	n=1 (3.3%)	n=1 (5.9%)	n=6 (42.9%)	0.002
Pulmonary Rales			n=15 (75%)	n=23 (56.1%)	0.153	n=14 (46.7%)	n=12 (70.6%)	n=12 (85.7%)	0.032

Chest X-ray findings:	No specific findings		n=3 (15%)	n=17 (41.5%)		n=13 (43.3%)	n=6 (35.3%)	n=1 (7.1%)	< 0.001
	Cardiomegaly		n=1 (5%)	n=11 (26.8%)		n=11 (36.7%)	n=0 (0.0%)	n=1 (7.1%)	
	Vascular cephalization		n=4 (20%)	n=5 (12.2%)	0.004	n=3 (10%)	n=4 (23.5%)	n=2 (14.3%)	
	Interstitial edema		n=8 (40%)	n=7 (17.1%)		n=3 (10%)	n=6 (35.3%)	n=6 (42.9%)	
	Alveolar edema		n=4 (20%)	n=1 (2.4%)		n=0 (0.0%)	n=1 (5.9%)	n=4 (28.6%)	

IVC collapsibility			19.61±4.63	24.77±6.03	<0.001	27.57±4.35	20.60±3.72	16.46±3.11	0.019
ProBNP(pg.\ml)			8400± 4086.6	5310± 2627.7	0.002	4171.67± 1658.92	7158.82± 2732	9918.57± 3832.36	< 0.001
PaO_2_/FiO_2_ ratio			288.40±47.51	313.37±42.73	0.043	327.40±26.83	303.82±37.03	259.21±53.40	< 0.001

### Assessment of sensitivity and specificity of RRI and VFP to predict cardiorenal syndrome

During the hospital stay, we discovered that the RRI and VFP are more correlated to renal functions after deterioration than the baseline renal function as strong evidence of its role in the prediction of cardiorenal syndrome early like the following: RRI did not show correlation to BUN with (P-value of 0.063) but correlated to maximum BUN (P-value of 0.005), to creatinine (P-value of 0.001), to maximum creatinine (P-value of < 0.001), to GFR (P-value of 0.006), to lowest GFR (P-value of 0.014) and to AKI (P-value of 0.031). VFP is correlated to BUN with (P-value of 0.047), to maximum BUN (P-value of 0.001), to creatinine (P-value of 0.002), to maximum creatinine (P-value of < 0.001), to GFR (P-value of 0.011), to lowest GFR (P-value of < 0.001) to AKI (P-value of < 0.001) and to hemodialysis need (P-value of 0.049).

**Fig. 2.**
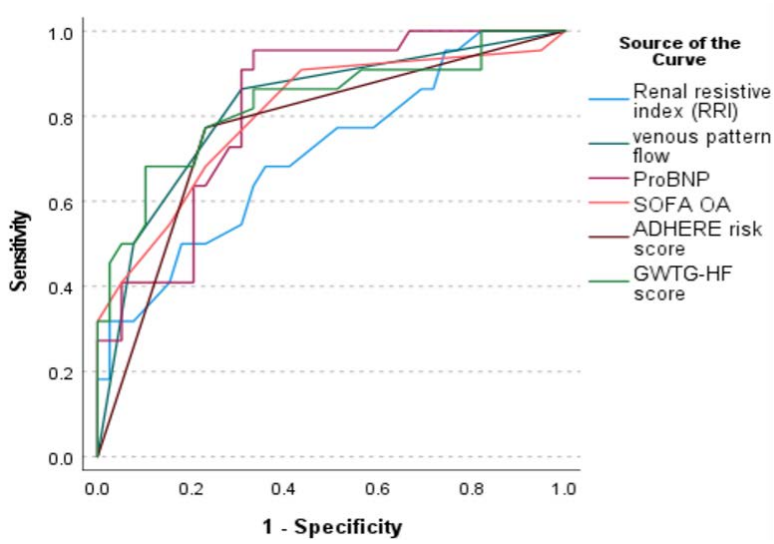
ROC curve for prediction of Acute Kidney Injury using RRI, venous flow pattern, proBNP, SOFA, GWTG, and ADHERE

The Receiver operating characteristic (ROC curve) of the RRI, VFP, proBNP, SOFA score, ADHERE risk score, and GWTG-HF scores for AKI occurrence in Figure 2 showed that RRI has 68% sensitivity and 64% specificity to detect AKI with an AUC of 0.711, but VFP has better results compared to RRI and other prognostic scores, with 86.4% sensitivity and 69.2% specificity with an AUC of 0.822.

**Fig. 3.**
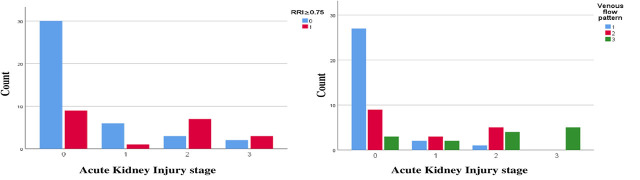
The classification of each stage of AKI based on RRI and the classification of each stage of AKI according to the VFP (RRI≥0.75 (0=no, 1=yes) VFP (1=Continuous, 2= biphasic, 3=monophasic)

Figure 3 shows the classification of each stage of AKI according to RRI and VFP. As in stages 2 and 3, the RRI ≥0.75 is more prevalent. Stage 2 of AKI showed mostly biphasic and monophasic patterns, while in stage 3, there is only a monophasic pattern.

### Assessment of sensitivity and specificity of the RRI and VFP to predict morbidity and mortality

At first, the beneficial outcome of RRI and VFP in prediction the morbidity (need for non-invasive ventilation, invasive mechanical ventilation, and failure of NIV to shift to IMV) of acute heart failure was assessed and the incidence of these endpoints in patients with RRI ≥0.75 and RRI <0.75 were as follows: the need of NIV 30%% versus 7.3 %, (p-value of <0.048); Invasive mechanical ventilation 5% versus 4.9%, (p-value of = 1); failure of NIV 5% versus 0%, (p-value of = 0.32), but VFP showed better results (P-value of 0.004) for NIV and (P-value of 0.010) for IMV but (P-value of 0.508) for prediction of failure of NIV. During hospital stay, VFP was better for prediction of in-hospital mortality as its incidence in each group was a monophasic pattern of 42.9%, biphasic pattern of 11.8%, and continuous pattern of 0 % (P-value of < 0.001), but RRI showed a poor correlation to the incidence of in-hospital mortality. During follow-up later for 3 months, the incidence of the composite endpoints in patients with RRI ≥0.75 and RRI <0.75 was as follows: hospital readmission 65.0% vs. 17.1% (P-value of < 0.001), 3-months mortality 35.0% vs. 9.8% (P-value of 0.030) but the incidence of these events in patients with continuous, biphasic or monophasic VFP were as follows: hospital readmission (10.0% vs. 52.9% vs. 57.1% respectively) P-value of < 0.001, 3-months mortality (3.3% vs. 29.4% vs. 35.7% respectively) P-value of 0.005. Also, RRI showed a correlation with SOFA score (P-value of < 0.001), ADHERE risk score (P-value of 0.002), and GWTG-HF score (P-value of 0.005), VFP correlates with the same scores as follows: SOFA score (P-value of < 0.001), ADHERE risk score (P-value of < 0.001) and GWTG-HF score (P-value of 0.002), as shown in Table 3.

**Table 3.** Comparisons between morbidity and mortality-related data after its classification according to RRI and VFP

	**RRI>=0.75**			**venous flow pattern**			
	
	**Yes (n=20)**	**No (n=41)**	**P-value**	**Continuous (n=30)**	**Biphasic (n=17)**	**Monophasic (n=14)**	**P-value**
Noninvasive ventilation	n=6 (30.0%)	n=3 (7.3%)	0.048	n=1 (3.3%)	n=2 (11.8%)	n=6 (42.9%)	0.004
Invasive mechanical ventilation	n=1 (5.0%)	n=2 (4.9%)	1	n=0 (0.0%)	n=0 (0.0%)	n=3 (21.4%)	0.010
failure of NIV to IMV	n=1 (5.0%)	n=0 (0.0%)	0.328	n=0 (0.0%)	n=1 (5.9%)	n=0 (0.0%)	0.508
length of hospital stays (days)	7.40±2.56	6.05±2.34	0.045	5.37±1.71	6.82±2.40	8.502.71	0.165
In-hospital mortality	n=4 (20.0%)	n=2 (4.9%)	0.084	n=0 (0.0%)	n=1 (5.9%)	n=5 (35.7%)	<0.001
Survival time within 3 months-follow up (days)	53.35±36.8	83.90±18.7	0.002	89.10±4.9	73.18±28.6	42.14±36.7	< 0.001
Readmission within 3 months	n=13 (65%)	n=7 (17%)	< 0.001	n=3 (10.0%)	n=9 (52.9%)	n=8 (57.1%)	< 0.001
3-months mortality	n=7 (35.0%)	n=4 (9.8%)	0.030	n=1 (3.3%)	n=5 (29.4%)	n=5 (35.7%)	0.005
SOFA score	4.85±3.01	1.95±1.76	<0.001	1.50±1.22	2.94±1.71	5.86±3.25	<0.001
ADHERE risk score	1.70±0.47	1.29±0.46	0.002	1.23±0.43	1.47±0.51	1.79±0.43	<0.001
GWTG-HF score	50.15±12.47	41.02±10.9	0.005	38.13±7.36	42.18±7.78	58.86±12.92	0.002

**Fig. 4.**
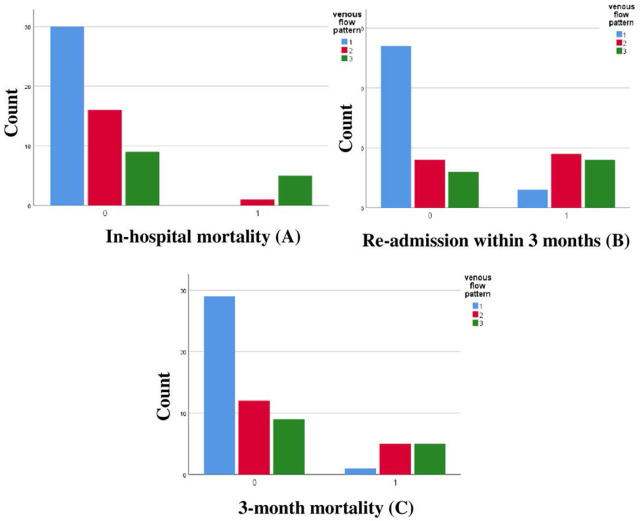
Classification of the incidence of in-hospital mortality (A), the incidence of readmission to hospital within 3 months (B), and 3-month mortality (C) according to VFP (1=Continuous, 2= biphasic, 3=monophasic)

**Fig. 5.**
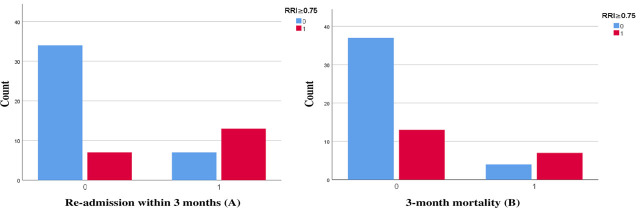
Classification of the incidence of readmission to hospital within 3 months (A) and the incidence of 3-month mortality (B) according to RRI (RRI≥0.75 (0=no, 1=yes)

This study’s findings showed that RRI has a prognostic role in the prediction of in-hospital mortality in acute heart failure as RRI has 83.3% sensitivity and 65.5% specificity with a cut-off value of 0.788, AUC of 0.788 and P-value of 0.001, VFP showed better results with 83.3% sensitivity and 83.3% specificity with AUC 0.880 and P-value of < 0.001 that was better than ADHERE risk score with only 61.8% Specificity. RRI also proved its role in predicting readmission to hospital for 3 months post-discharge with 65% sensitivity and 82.9% specificity with AUC 0.748 and P-value of < 0.001, while VFP also showed 85% sensitivity and 65. % specificity with AUC 0.76 and P-value of < 0.001. The results here showed that VFP has a better predictive value for the incidence of 3 months mortality with 90.9% sensitivity and 58% specificity with AUC 0.758 and P-value of < 0.001, while RRI has 63.4% sensitivity and 74 % specificity with AUC 0.701 and P-value of 0.023. Figure 4 represents the classification of in-hospital mortality, the incidence of hospital readmission within 3 months, and the 3-month mortality incidence according to VFP. Figure 5 shows the classification of in-hospital mortality, the incidence of hospital readmission within 3 months, and the 3-month mortality incidence according to RRI.

## Discussion

ADHF is a significant contributor to hospital admissions and mortality, with substantial social and economic implications. A major clinical challenge in managing heart failure lies in accurately identifying patients who are at the most significant risk of adverse outcomes. Depending on how the patient responds to treatment, this risk can fluctuate throughout a hospitalization. Despite this, no practical risk stratification tools are available to assess patients during the critical period following a decompensation event. This phase represents a vulnerable point in the disease’s progression, where the likelihood of rehospitalization and mortality is significantly heightened.

The relationship between cardiac and renal diseases has garnered significant attention recently. The impact of deteriorating renal function (WRF) on prognosis in patients with ADHF remains contentious, with substantial evidence supporting both perspectives. Numerous research studies [32,33,34] indicate a substantial correlation between WRF during hospitalization and negative results post-discharge; nevertheless, other studies demonstrate that, despite the common occurrence of WRF, there is no evidence of poorer clinical outcomes [35,36,37].

The development of duplex ultrasound has enabled the evaluation of changes in patients’ renovascular resistance and intra-renal blood flow. This technique is noninvasive and may be repeated as often as required. The parameters measured in the intra-renal duplex include RRI and venous flow patterns assessment. In different clinical settings, the RRI has been demonstrated to be associated with a greater probability of renal disease progression and with a worse prognosis [38].

The measurement of RRIs is typically aided by the non-critical nature of the insonation angle setting for their sampling. Measurement imperfections, such as errors in estimating peak systolic velocity and end diastolic velocity, affect the formula’s numerator and denominator equally, thereby compensating for one another [38,39]. Intra-observer variability varied from 2.07% to 5.1%, whereas inter-observer variability ranged from 3.61% to 6.2% [40]. Our study sought to investigate the possible impact of alterations in intra-renal duplex parameters in predicting worsening renal function and death in patients with acute decompensated heart failure.

The findings of this study revealed a strong correlation between VFP and all studied variables, including NYHA class, central venous pressure (CVP), lower limb edema, the presence of a third heart sound, pulmonary rales, proBNP levels, PaO_2_/FiO_2_ ratio, chest X-ray findings, and inferior vena cava (IVC) collapsibility index. In contrast, RRI showed correlations with CVP, chest X-ray findings, IVC collapsibility index, proBNP levels, and PaO_2_/FiO_2_ ratio. Additionally, the study observed that higher RRI values (>0.75) combined with severe venous congestion (grade 3 monophasic pattern) were associated with elevated proBNP levels, indicating significant volume overload. Conversely, high RRI values (>0.75) with milder venous congestion were linked to lower proBNP levels, suggesting reduced congestion.

Grande et al. [41] highlighted the reasons why VFP is superior to RRI in assessing volume status. They explained that multiple factors beyond arteriolar tone, such as arterial stiffness, atherosclerosis, parenchymal damage, and renal venous congestion influence arterial resistance. Additionally, prolonged vasoconstriction can reduce the number of perfused vessels without causing anatomical changes in the microvascular structure. This phenomenon, known as** vascular rarefaction** [42], can lead to progressive vascular remodeling due to tissue ischemia, the release of enzymes, and growth factors, ultimately resulting in fibrosis [43]. On the other hand, right atrial function and the histological characteristics of the surrounding renal parenchyma influence the flow pattern in intrarenal veins. Jeong et al.’s [44] study further supported this observation, confirming intrinsic renal factors’ role in shaping venous flow patterns.

By reviewing the literature, different definitions for WRF were found to be used in clinical trials, but the most widely used and accepted definition of WRF has been an increase in serum creatinine ≥0.3 mg/dL from baseline [45], as we used in our study. The percentage of patients who develop WRF in the different studies ranged from 20% to 50%. Maeder et al. [46] studied 566 patients included in the TIME-CHF trial and reported that WRF, characterized by an elevation in serum creatinine by 0.3 mg/dl from the baseline, occurred in 40% of the patients [46].

This study’s findings demonstrated a moderate correlation between RRI and the development of AKI but a strong correlation between VFP and AKI. Both showed good sensitivity and specificity in predicting worsening renal function, as atherosclerosis and nephropathy, exacerbated by concurrent hypertension and diabetes, primarily elevate intrarenal arterial resistance and diminish compliance. Consequently, the diverse parameters linked to cardiovascular disease may render RRI less precise in evaluating renal congestion in heart failure compared to the IRVF profile.

The superiority of VFP over RRI in predicting AKI might be attributed to the direct reflection of VFP on hemodynamics [47]. Employing Doppler modalities in ultrasound examinations, venous flow patterns in relation to the cardiac cycle can be evaluated [47]. Hemodynamic alterations in the systemic venous circulation resulting in elevated venous pressure are associated with anomalies in the venous Doppler profile at various locations. Clinically substantial systemic venous congestion is identified through aberrant Doppler flow patterns. The venous excess ultrasound (VExUS) score was evaluated in patients undergoing right cardiac catheterization, revealing a substantial correlation between correct atrial pressure and VExUS grade [48]. Organ congestion due to venous hypertension may contribute to organ harm in various clinical scenarios, including acute illnesses, congestive heart failure, and chronic kidney disease [49]. In 2023, four studies examined the correlation between AKI and venous congestion. Post-cardiac surgery, irregularities in intra-renal venous flow, portal vein pulsatility fraction, hepatic vein flow patterns, and central venous pressure were linked to the onset of AKI [50].

Several studies have examined the influence of RRI in forecasting WRF in heart failure patients. Lacoviello M. et al. [51] investigated the function of RRI in forecasting WRF in outpatients with chronic heart failure undergoing conventional therapy, revealing that RRI was correlated with WRF. Nicolas Bihry et al. [22] reported that RRI was correlated with age, creatinine, and cystatin C but not with other clinical or echocardiographic factors [22]. Also, de la Espriella et al. [47] discovered that discontinuous IRVF patterns were associated with increased odds of WRF [52].

On the other side, confounding factors like hemodynamic stability and medication use are to be considered. For instance, Chen et al. [53] reported discrepancies between proximal renal venous flow (PRVF) and intrarenal venous flow (IRVF) patterns in 31.9%, with PRVF patterns exhibiting greater severity in 88% of these cases [53]. A notable association was identified between PRVF and CVP, while this trend was less pronounced in IRVF. It was suggested that the patterns of PRVF and IRVF are not completely aligned; a systematic assessment is beneficial for identifying the intervention site for renal vein reflux diseases [53].

We also tried to classify the AKI stage according to RRI and VFP and found that the mean RRI isn’t a good discriminator for AKI stage 1 from the non-AKI group, but in stages 2 and 3, the mean RRI is much higher than the non-AKI group. Also, Stage 2 of AKI showed mostly biphasic and monophasic patterns, while in Stage 3, there was only a monophasic pattern. That was also studied by Jeong et al. [44]; it was determined that the RRI was not an effective discriminator for AKI stage 1, but patients with AKI stages 2 and 3 had a considerably elevated RRI compared to those without AKI [44]. We also found that we can use VFP as a predictor for the need for hemodialysis, but the number of inclusions for this need was just two patients, which is just a small number to confirm this result.

Our study’s RRI and VFP were good predictors for in-hospital and post-3-month composite outcomes, as VFP was a good predictor for the need for ventilatory support and in-hospital mortality. Both RRI and VFP were strong predictors for readmission and 3-month mortality. There are multiple contradictory data in the literature regarding this issue. In the clinical context of heart failure, the RRI was assessed in a cohort of patients with heart failure and retained ejection fraction. Ennezat et al. [54] reported that renal impairment was elevated in patients with heart failure compared to those with just arterial hypertension, and it was correlated with a poor prognosis. Ciccone et al. [38] investigated the significance of RRI in forecasting adverse outcomes in patients with decreased ejection fraction heart failure. RRI was independently linked to a composite endpoint comprising hospitalization for ADHF and mortality resulting from exacerbated heart failure.

Noriko Iida et al. [55] endorsed the notion that VFP is superior to RRI in forecasting acute heart failure outcomes, as IRVF patterns, rather than RI, are contingent upon RAP, indicating a relationship with renal congestion. Furthermore, IRVF patterns exhibited a substantial correlation with clinical outcomes, independent of RAP and other risk variables, and may offer supplementary information for stratifying at-risk HF patients. Rola et al. [56] directly measured intrarenal vein pressure in their research of various patients, rather than utilizing central venous pressure (CVP). Discontinuous IRVF, especially the monophasic pattern and the enhanced VExUS grading system, offered supplementary insights for a thorough assessment of venous congestion or fluid status and informed decisions on prompt fluid removal or cessation of fluid resuscitation [56].

Puzzovivo et al. [57] findings indicated the independent and incremental significance of Doppler venous patterns indicative of renal congestion in forecasting heart failure progression in individuals with congestive heart failure [57]. Wallbach et al. [58] reported a greater incidence of clinical events (mortality or requirement for renal replacement therapy) in patients exhibiting monophasic and biphasic IRVF patterns, in contrast to those with pulsatile and continuous IRVF patterns [58]. Komuro et al. [59] identified significant yet weak correlations of RRI with serum creatinine and estimated glomerular filtration rate. Additionally, the RRI reflects the deterioration of intrarenal hemodynamics that cannot be sufficiently explained by eGFR alone. The authors highlighted that the evaluation of the RRI may be beneficial with prognostic assessments for individuals with cardiovascular disease [59].

Our findings offer important insights that can be compared to existing clinical guidelines for the management of ADHF. Firstly, The RRI’s correlation with CVP as a marker of volume overload aligns with current guidelines that emphasize the importance of evaluating fluid status in ADHF. Guidelines recommend using clinical signs, such as jugular venous distension and peripheral edema, alongside objective measures like CVP to assess volume status [60]. Additionally, as prognostic factors, our results highlighted that both RRI and VFP correlate significantly with renal function markers, such as BUN and creatinine. This is consistent with guidelines that stress the need for monitoring renal function in patients with ADHF, particularly given the risk of cardiorenal syndrome [61].

Furthermore, the strong correlation of RRI and VFP with proBNP levels supports existing recommendations for using biomarkers in diagnosing and managing heart failure. Current guidelines advocate for measuring BNP or proBNP to aid in the diagnosis and assess prognosis in heart failure patients [60]. Also, our study’s findings that VFP demonstrates superior predictive value for in-hospital mortality and readmission compared to RRI align with the guidelines’ emphasis on identifying patients at high risk for adverse outcomes. Using these parameters to stratify risk can enhance clinical decision-making and guide therapeutic interventions [62]. Finally, the correlation of RRI and VFP with scores like SOFA and ADHERE emphasizes the guidelines’ recommendation to utilize comprehensive assessment tools to evaluate heart failure severity and prognosis [61].

### Study Limitations

This research possesses certain limitations. First of all, the relatively small number of patients limits the generalizability to larger and more diverse populations. Further studies with a larger sample size are warranted. The study was a retrospective, single-center, observational investigation, so the results might not be generalizable to other hospitals with different patient populations. There might be a potential inter-observer variability in measuring RRI and VFP. The influence of both identifiable and unidentifiable confounders must not be overlooked. Owing to the restricted quantity of composite outcomes, merely two patients necessitated hemodialysis. We did not evaluate microalbuminuria, cystatin C, or other biomarkers indicative of renal dysfunction. Other unmeasured confounders, such as diuretics, ACE inhibitors, and beta-blockers, can impact renal hemodynamics. The evaluation of cardiac and renal hemodynamics using direct pressure measurements via right-sided catheterization was not performed. Estimates of several metrics, including CVP, may lack accuracy [63]. Aortic valve dysfunction may influence RRI values; however, subgroup analysis was not conducted due to the limited patient population.

## Conclusion and recommendations

Comprehending the variations in cardio-hemodynamics during ADHF enhances diagnostic and prognostic precision. Evaluating RRI and VFP is crucial in determining volume status in ADHF. Renal duplex measures are effective prognostic instruments for deteriorating renal function, with a sensitivity of 86.4% and specificity of 69.2% for VFP and a sensitivity of 68% and specificity of 64% for RRI, respectively. Renal duplex is a straightforward, non-invasive instrument that identifies several hemodynamic parameters at admission and difficulties throughout hospitalization. Renal duplex can be utilized as a predictive instrument for mortality and survival in acute decompensated heart failure, potentially altering the initial therapeutic strategy for these patients.

The paper suggests that intra-renal duplex parameters (RRI and VFP) should be evaluated in hospitalized patients with ADHF to assess volume status and predict worsening renal function. Standardized protocols for measuring RRI and VFP should be established, focusing on specific ultrasound settings, measurement sites (preferably at the corticomedullary junction), and patient positioning to ensure consistency and accuracy in results [64]. Incorporating RRI and VFP measurements in routine clinical practice can improve renal hemodynamics assessment and patient management, especially in critical care settings [65]. Doppler ultrasound, a noninvasive tool, is crucial for measuring RRI and VFP, and regular monitoring can help address changes in renal perfusion and treatment response [66]. Technical and interpretation training for clinicians is also recommended [64,65,66].
